# Performance Comparison of Xpert HIV-1 Viral Load Assay and Roche Taqman and Abbott M2000 RT in Bamako, Mali

**Published:** 2020-07-30

**Authors:** Bourahima Kone, Drissa Goita, Oumar Dolo, Daouda Traore, Dramane Sogoba, Amadou Somboro, Moumine Sanogo, Anou M Somboro, Nadie Coulibaly, Alou Sanogo, Zoumana Diarra, Madou Traore, Almoustapha I Maiga, Bocar Baya, Yeya Dit Sadio Sarro, Bassirou Diarra, Amadou Kone, Dramane Diallo, Djeneba Dabitao, Jane L. Holl, Michael Belson, Sounkalo Dao, Robert L. Murphy, Mahamadou Diakite, Souleymane Diallo, Seydou Doumbia, Mamoudou Maiga

**Affiliations:** 1 University Clinical Research Center (UCRC)-SEREFO, University of Sciences, Techniques and Technologies of Bamako (USTTB), Bamako, Mali; 2 Centre d’Ecoute, de Soins, d’Animation et de conseils (CESAC), Bamako, Mali; 3 Point-G University Teaching Hospital, Bamako, Mali; 4 Sikasso, Regional Hospital, Mali; 5 National Institute of Allergic and Infectious Diseases (NIAID), Bethesda, Maryland, USA; 6 Center for Innovation in Global Health Technologies (CIGHT), Northwestern University, Evanston, Illinois, USA; 7 Mali National Institute of Public Health (INSP), Bamako, Mali; 8 University of Chicago, Illinois, USA

**Keywords:** Viral load, Xpert HIV-1, Roche TaqMan, Abbott m2000 RT, Mali

## Abstract

**Background:**

Routine monitoring of HIV-1 Viral Load (VL) is important in patients on Antiretroviral Therapy (ART) management. Access to HIV VL remains a challenge in resource-limited settings, especially in rural areas. Universal access to VL requires more simplified and less restrictive alternatives to current conventional VL methods. The objective of this study was to evaluate the performance of the new rapid (2-hour turnaround time) Xpert HIV-1VL technique compared to Roche TaqMan and Abbott RT m2000 for HIV-1 RNA quantification in HIV- infected patients.

**Study design:**

We conducted a cross-sectional study in patients seen for routine VL monitoring between August and November 2018 in a HIV care site in Bamako. The performance of the Xpert HIV-1 VL assay was evaluated against the Roche TaqMan assay and Abbott m2000 RT assay. Performance, utility and reliability/reproducibility were verified using accuracy, sensitivity, specificity, positive and negative predictive values, Diagnostic Odds Ratio (DOR), Kappa coefficient, Pearson correlation coefficient, and Bland-Altman analysis.

**Results:**

The Xpert assay compared well with the two current referral assays (Roche TaqMan and Abbott m2000 RT assays). Compared to Roche TaqMan assay the sensitivity was 93.10%, specificity (97.01%) and accuracy (95.20%), the correlation coefficient of Pearson (r) was 0.98 (p <0.01). Bland-Altman analysis showed a mean difference of 0.18 log10 cp/mL; (Standard Deviation) SD=0.33. Compared to the Abbott m2000 RT, the sensitivity, the specificity and the accuracy were respectively 93.44%; 92% and 92.65%. The Xpert HIV-1 VL assay showed a good correlation with a correlation coefficient of Pearson, r=0.99 (p <0.001). The overall mean difference in the HIV-1 VL values obtained by Xpert HIV-1 VL and Abbott m2000 RT assays was 0.08 log10 cp/mL; SD=0.30.

**Conclusion:**

Xpert HIV-1 VL showed a good performance compared to Roche TaqMan and Abbott m2000 RT. With the rapid test results (less than 2 h) and ease of testing individual specimens, the Xpert HIV-1 VL assay could be an effective alternative for HIV VL monitoring in resource-limited settings.

## Introduction

In 2017, an estimated number of 36.9 million people worldwide were living with HIV infection; 95% of which were infected with HIV-1, and only 21.7 million were receiving Antiretroviral Therapy (ART) [1]. However, with free access to antiretroviral therapy and the recent recommendations by the World Health Organization to start ART with patients diagnosed infected with HIV-1, regardless of CD-4 count [2,3]. The number of people receiving ART and requiring Viral Load (VL) monitoring is expected to increase substantially. In addition, the 90-90-90 goals of the United Nations Program on HIV/AIDS (UNAIDS) seeks to achieve viral suppression in 90% of individuals receiving ART by end of 2020 [4,5]. Around 50% of people living with HIV are in Low and Middle Income Countries (LMICs) with limited resources for VL testing equipment and supplies.

Monitoring of HIV-1 VL is a cornerstone of HIV-1 management, globally. HIV-1 VL assay is performed to monitor the efficiency of ART after the initiation of treatment, to identify rapidly treatment failure, to target adherence counselling, and to guide decisions about switching antiretroviral regimens [6–17]. According to the WHO, VL monitoring is the preferred approach to wisely diagnose and confirm treatment failures, which they strongly recommend [18–20].

In practice, routine VL testing has been a challenge, particularly in low- and middle-income countries due to the required sophisticated and costly equipment, as well as trained technicians to perform the assays [16,17,20,21]. As consequences of the difficult access to VL in these areas, are undiagnosed failure of treatment, late treatment switches, and potential occurrence and spread of HIV drug resistance [20].

Recent technologies for HIV-1 VL detection and quantification measure Reverse Transcriptase (RT) enzymatic activity, and most commonly, utilize molecular methods for example transcription-mediated amplification, sequence-based amplification of nucleic acids or Real-Time Polymerase Chain Reaction (RT-PCR). These molecular techniques employ high-capacity platforms through the combination of separate system of extraction and amplification of the viral genome [17]. Most current HIV-1 VL platforms require expensive laboratory infrastructure, technical expertise, maintenance of the instruments, and well-trained technicians, making widespread VL testing unaffordable and impractical in most LMICs [17,21,22]. Low-cost, simple, easy-to-perform, and rapid HIV VL testing platforms are urgently needed in resource-limited countries.

The GeneXpert technology, developed originally for other types of infectious diseases, such as hepatitis C virus, mycobacterium tuberculosis, and methicillin-resistant Staphylococcus aureus, uses cartridges in distinct modules to operate the extraction of the genetic material and their quantitation through RT-PCR. GeneXpert instrument system served as model for the Xpert-HIV-1 VL and this could be a rational alternative to the costlier and more complex HIV-1 VL testing platforms based on the simplicity and ease of use of this assay it [5,17,21].

This current work seeks to assess the diagnostic performance and the accuracy of the Xpert-HIV-1 VL technique to identify and quantify HIV-1 RNA relative to two of the most commonly used testing platforms; the Roche TaqMan and the Abbott m2000 RT assays.

## Materials and Methods

### Type of study and data collection

The cross-sectional study was performed, using specimens collected from August 2018 to November 2019. Patients with HIV-1 under ART treatment and HIV-1 positive subjects not yet started treatment, monitored by the Department of Infectious Diseases at the Point-G University Teaching Hospital and the “Centre d’Ecoute, de Soins, d’Animation et de Conseils” (CESAC), in Bamako, Mali, were consecutively enrolled in this study.

The evaluation was performed in the HIV/TB Research and Training Center (SEREFO) laboratory of the University Clinical Research Center (UCRC/SEREFO), in Bamako, Mali. Since 2005, the UCRC/SEREFO laboratory participated in External Quality Control (EQC) by the American College of Pathologists (CAP-VL) for the Roche TaqMan and CDC for Abott m2000rt Machine and demonstrated satisfactory performance.

### Inclusion and exclusion criteria

All patients infected with HIV-1, either on ART or not yet started on ART, were eligible for inclusion. Were excluded patients infected HIV-2 and those who were co-infected with HIV-1 and HIV-2.

### Laboratory tests

All samples were blindly tested on the three different molecular platforms: (1) GeneXpert (Cepheid, Sunnyvale, United States), (2) Roche TaqMan (Roche Molecular Systems, Branchburg, USA) and (3) Abbott m2000rt (Abbott Laboratories, Matsudo-Shi, Chiba, Japan). The following commercially available Kits including primers sequences were used to perform the HIV viral load test: the Xpert® HIV-1 Viral Load kit (Cepheid: 632 E Caribbean Dr, Sunnyvale, CA 94089, United States), the COBAS® AmpliPrep/COBAS® TaqMan® HIV-1 Test v2.0 kit (Roche Molecular Systems, Inc. 1080 US Highway 202 south Branchburg, NJ 08876 USA) and Abbott m2000rt kit (Abbott GmbH & Co.KG Max-planck-Ring 2 65205 Wiesbaden, Germany).

The Xpert-HIV-1 VL assay was conducted based one to the instructions of the manufacturer. Briefly, 1mL of plasma sample was added into the cartridge and charged into the GeneXpert instrument. The total RNA extraction process, purification, reverse transcription and cDNA quantitation were achieved within the fully automated cartridge system.

The COBAS® AmpliPrep/COBAS® TaqMan® HIV-1 method, couples an automated Nucleic Acids (NA) isolation system on the COBAS® AmpliPrep Instrument with automated amplification and detection on the COBAS® TaqMan® Analyzer, employing hydrolysis probes technology. The reverse transcription, amplification primers and the probe are targeted at a sequence within the highly conserved region of the HIV-1 gag gene. To perform the experiment, a volume of 850 uL of EDTA plasma were used in an automated sample preparation procedure on the COBAS® AmpliPrep Instrument by a generic silica-based capture method to isolate total NA. To the samples and controls, a Quantitation Standard (QS) was added at a known concentration along with magnetic glass beads and chaotropic lysis binding buffer that bind released NA. After the steps of separation and washing, the NA was then eluted in aqueous buffer, and the eluate added automatically to the master mix. The PCR tubes were automatically transferred to the COBAS® TaqMan® Analyzer for amplification.

The Abbott-RT HIV-1 test was also conducted according to the manufacturer’s instructions. Briefly, the m2000sp automated extractor served to obtain a purify RNA from a 0.6 mL plasma sample and this was used for the quantitative real-time PCR (qRT-PCR) amplification and detection employing the fully automated m2000rt instrument.

### Data analysis

The Xpert-HIV-1 VL assay results were expressed in both copy cp/mL and log10 cp/ml, directly and the Roche TaqMan and Abbott m2000 RT assay results were expressed as copies (cp)/mL and were converted to log10 cp/mL. Log10 cp/mL values were used for analysis.

To assess Xpert-HIV-1.VL test performance, results were classified as (1) VL Not detected (< 1.60 log10 cp/mL for the Xpert-HIV-1 VL and the Abbott-m2000 RT methods and <1.30 log10 cp/mL for the Roche TaqMan assay) and (2) quantified VL (>1.60 log10 cp/mL for Xpert-HIV-1 VL and Abbott m2000 RT assays and >1.30 log10 cp/mL for Roche TaqMan assay). Agreement between tests was determined by weighted Cohen’s kappa.

The Pearson’s correlation squared R2 and the Pearson correlation coefficient (r) values were calculated on the basis of a simple linear regression to assess the linear relationship between the different techniques. Bland-Altman analysis was employed to evaluate the agreement between the different techniques of VL quantification. T-Tests and p-values <0.05 were considered statistically significant.

### Ethical considerations

Ethical clearance was obtained from the Ethics Committee of the University of Sciences, Techniques, and Technologies of Bamako (USTTB) and the Institutional Review Board (IRB) of the NIH-NIAID. A written informed consent was obtained from each study subject.

## Results

A total of 138 study subjects consented to use of their samples and 10 external control samples were obtained from the College of American Pathology, for a total of 148 samples.

The clinical and demographic characteristics of the 138 study subjects are summarized in Table 1. Most subjects were female (68%) and between 18–35 years old (37%).

Most subjects were receiving ART (68%) comprising of a nucleoside Reverse Transcriptase Inhibitor (NRTI) and a Non-Nucleoside Reverse Transcriptase Inhibitor (NNRTI).

Of the 148 samples tested, 125 were successfully tested by both the Xpert-HIV-1 VL and Roche TaqMan methods and 136 samples were successfully tested by both the Abbott m2000 RT and Xpert-HIV-1 VL techniques.

### Comparison of Xpert-HIV1-VL to Roche TaqMan

A total number of 125 samples with valid assay results for both of the Xpert-HIV-1 VL and Roche TaqMan assays were compared. By using the quantification thresholds of the two assays, the VL results were classified as “not detected” or “quantified” (Table 2a). Sensitivity was 93.10% [95% CI, 83.57% – 97.29%], specificity was 97.01% [95% CI, 89.75% – 99.18%], and the Cohen’s kappa was 0.9 (Table 2b). Accuracy was 95.20% [95% CI, 89.92% – 97.78%] with discordant results in 6 samples (4.80%), of which 2 were quantified by the Xpert-HIV-1VL method but not detected by the Roche TaqMan assay and 4 were quantified by the Roche TaqMan assay but not detected by the Xpert-HIV-1VL assay.

### Correlation between Xpert-HIV-1 VL and Roche TaqMan

**Figure 1.**
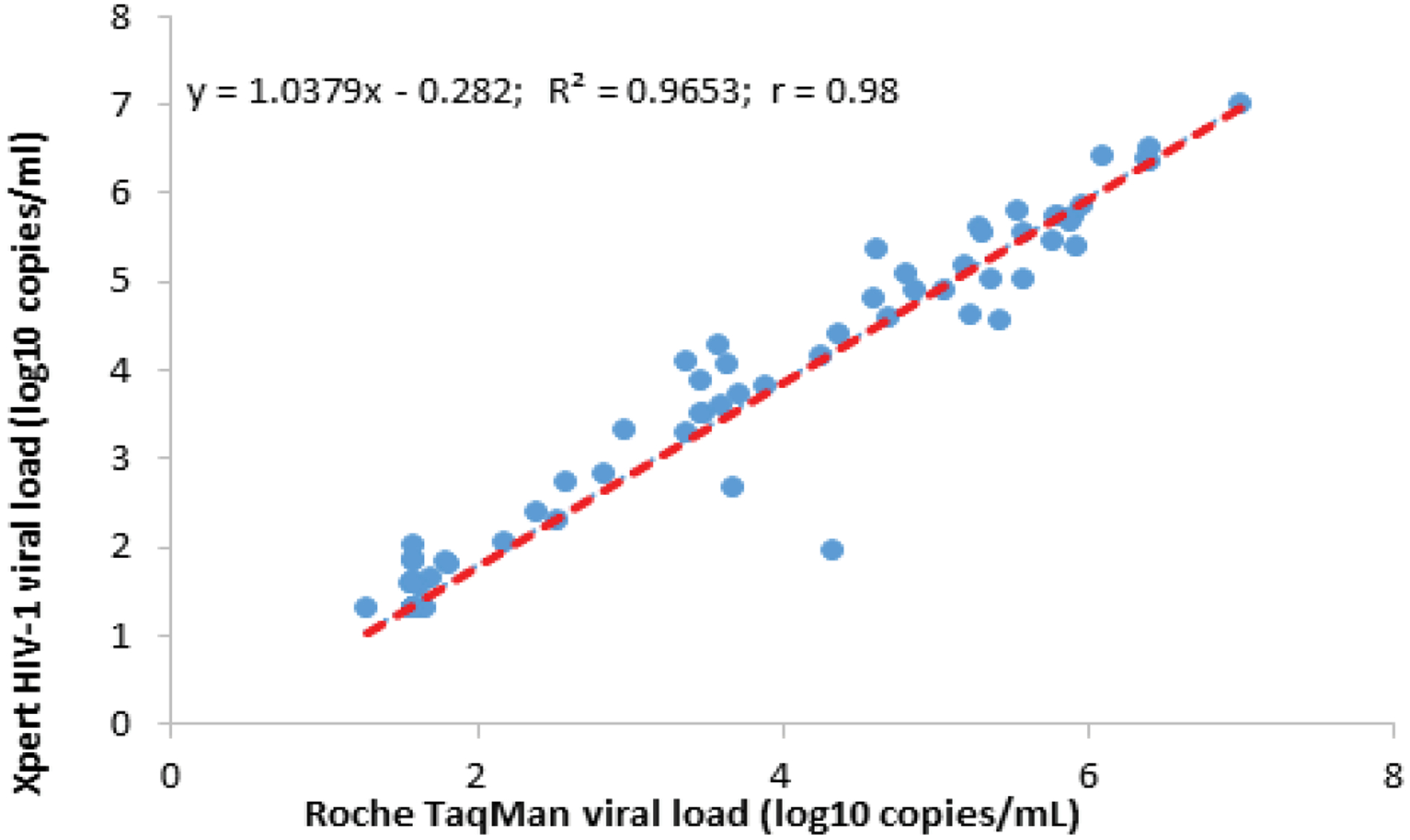
Scatter Plot of Xpert HIV-1 versus Roche TaqMan Viral Loads.

The mean VL obtained by the Xpert-HIV-1 VL method was 2.74 log10 cp/mL with a Standard Deviation (SD) of 1.67 log10 cp/mL, while the mean VL obtained by the Roche TaqMan assay was 3.56 log10 cp/mL (SD: 1.76 log10 cp/mL), with no statistically significant difference (p=0.41). A simple linear regression showed a significant linear correlation between the Xpert-HIV-1 VL and the Roche TaqMan assays, with a Pearson’s correlation coefficient of r=0.98 and a coefficient of determination, R2=0.9653 (p-value <0.001) (Figure 1).

**Figure 2.**
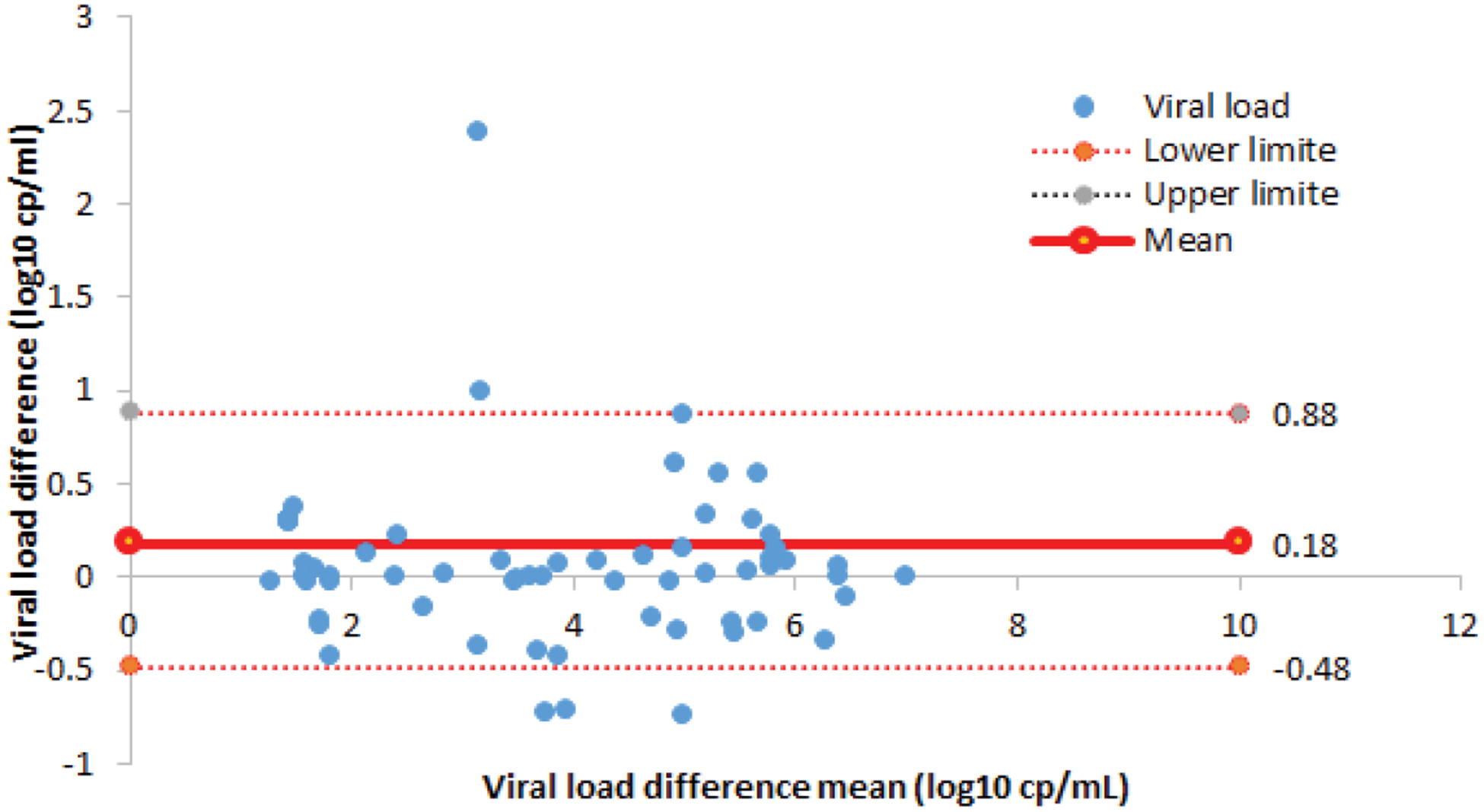
Bland-Altman Plot Concordance between Xpert HIV-1 and Roche TaqMan VL assays.

Using the Bland-Altman analysis, the mean difference of VL between the two assays was 0.18 log10 cp/mL (95% CI, − 0.48–0.88) with a SD of 0.33 log10 cp/mL (Figure 2).

### Comparison of Xpert-HIV-1 VL to Abbott m2000 RT

A total of 136 samples with valid results for both Xpert-HIV-1 VL and Abbott m2000 RT were compared. Using the quantification thresholds of the two assays, the VL results were classified as “not detected” if < 1.60 log10 cp/mL or “quantified” if ≥1.60 log10 cp/mL were detected (Table 3a). The sensitivity, specificity and kappa coefficient were respectively 93.44% [95% CI; 84.32–97.42], 92% [95% CI; 83.63–96. 28] and 0.85 [95% CI; 0.68–1.02] (Table 3b). In both assays, the HIV-1 VL was quantified in 57 samples and not detected in 69 samples, leading to overall accuracy of 92.65% (95% CI; 86.99%−95.96%). Discordant VL results were observed in 10 samples (7.3%). Four (4) sample quantified by Abbott m2000 RT assay was classified as not detected by Xpert HIV-1VL assay and six (6) samples were quantified by the Xper-HIV-1VL assay but classified as not detected by Abbott m2000 RT assay.

### Correlation between Xpert-HIV-1VL and Abbott m2000 RT

**Figure 3.**
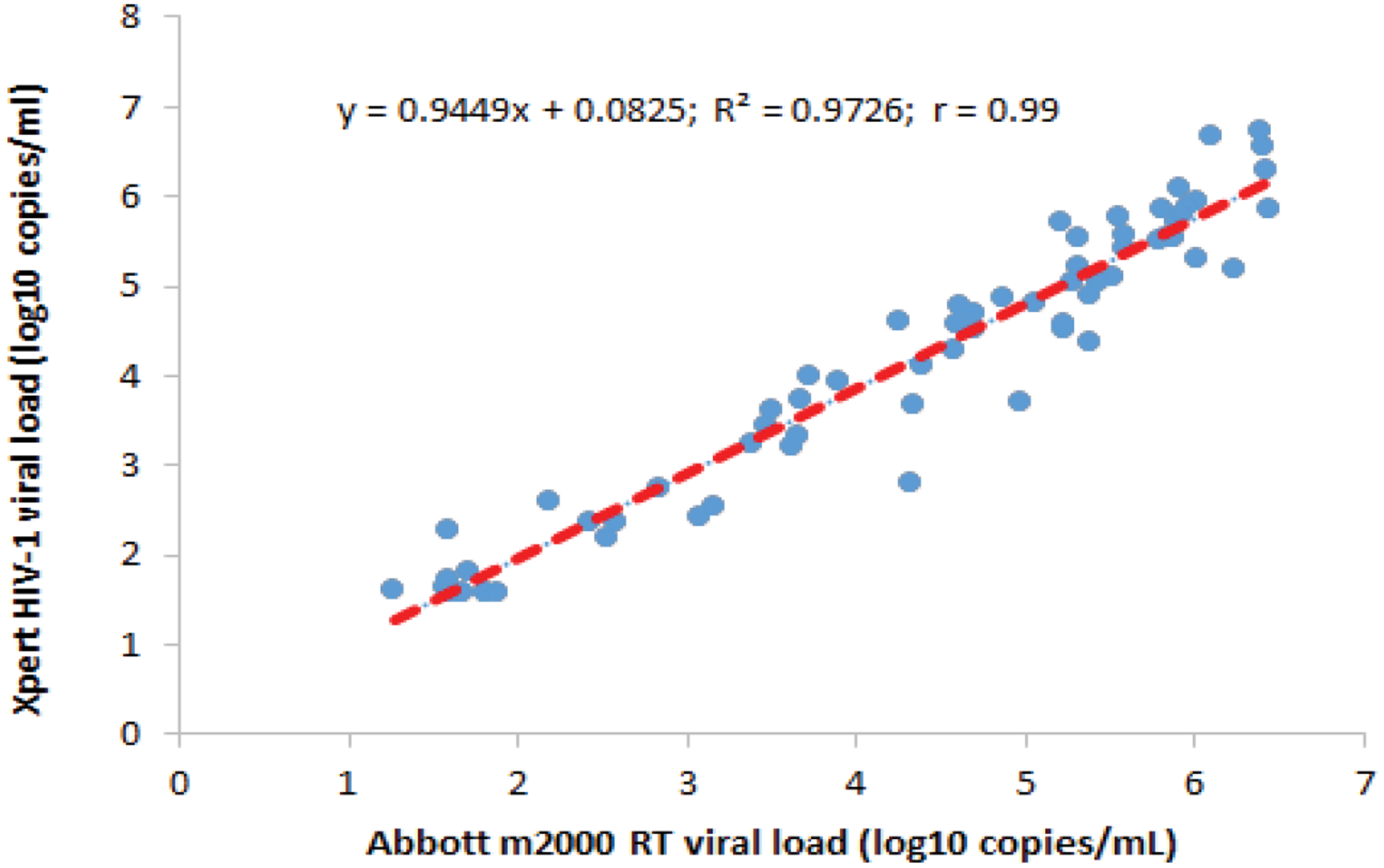
Scatter Plot of Xpert HIV-1 versus Abbot m200RT Viral Loads.

**Figure 4.**
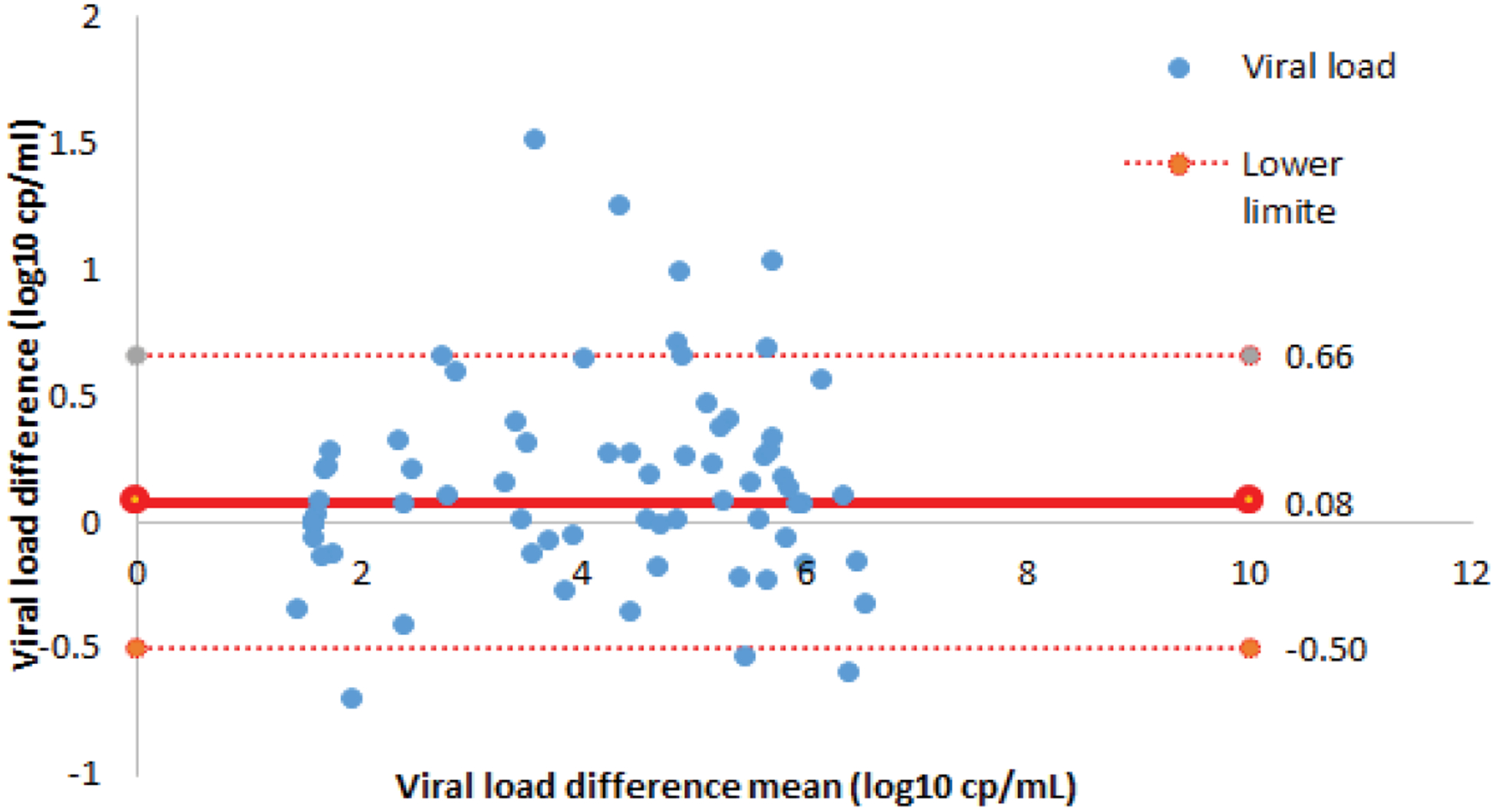
Bland-Altman Plot Concordance between Xpert HIV-1 and Abbot m2000RT Viral Load assays.

The mean VL obtained by the Xpert-HIV-1 VL and Abbott m2000 RT assays was 2.92 log10 cp/mL with a SD of 1.76 log10 cp/mL and 2.59 log10 cp/mL (SD 1.69 log10 cp/mL), respectively, with no statistically significant difference (p=0.71). A simple linear regression showed a significant linear correlation between the two assays with a Pearson’s correlation coefficient, r=0.99 and a coefficient of determination, R2=0.9726 (p-value <0.001). Figure 3 shows the scatter plot. Using the Bland-Altman analysis (Figure 4), the mean difference of VL between the two assays was 0.08 (95% CI, 0.50–0.66) with a SD of 0.30.

## Discussion

This study found, using for the first time, samples from HIV-1 infected patients in West Africa that the Xpert-HIV-1 VL assay performs as well as two well-established reference standard VL assays. We found comparable performance of the Xpert-HIV-1 VL assay with both the Roche TaqMan (sensitivity: 93.10%; specificity: 97.01%; agreement: 0.9; and accuracy: 95.02%) and Abbott m2000 RT.

Gous N et al. had similar findings when comparing the Xpert-HIV-1 VL and Roche TaqMan assays, with a Positive Predictive Value, PPV=86.7% (69.3–96.2) and a negative predictive value of 98.4% (94.3–99.8). In addition, only 2/5ths of the Roche TaqMan assay results were misclassified by the Xpert-HIV-1 VL method [9]. A recent study by Avidor B et al. also found that the sensitivity of the two methods is equivalent except for a CRF02_AG subtype variant with high VL titters, which was detected by the Roche TaqMan assay but undetected by the Xpert assay and a high coefficient of determination (R2=0.94) and a Pearson’s correlation coefficient (r=0.97) [5]. This study also found mean VLs by the Xpert-HIV-1VL and Roche TaqMan of 2.74 ± 1.67 log10 cp/mL and 2.56 ± 1.76 log10 cp/mL, respectively, which were not statistically significantly different (p=0.41). Similar results have also been reported [20].

The comparison results of the Xpert-HIV-1 VL and Abbott m2000 RT methods are similar to those of Kulkarni S et al., using samples from South Africa (sensitivity: 97%; specificity: 97%, and PPV: 99%; and a NPV: 89%). In this study, the Xpert-HIV-1VL test was also found to be highly sensitive (91% to 95%) and specific (99% to 100%) [18].

The single-use cartridge-based Xpert-HIV-1 VL method, by its simplicity and aptitude to deliver results in ~90 minutes (compared to several days for Roche TaqMan or Abbott m2000 RT assays), makes this technology well suited, particularly for use in low resource settings, such as Mali. The simplicity overcomes the paucity of highly skilled technicians and the ability to generate same day, point-of-care test results greatly improves time to and rate of ART initiation or change in ART regimen, by no longer requiring patients to return for another visit to obtain their results and treatment, many of whom have long travel times to reach a clinic. Furthermore, the Xpert HIV-1 VL assay can be even more cost-effective by batching multiple samples and is highly adaptable to different care settings, such as a large laboratory in a city, small clinic in a rural community, or even an emergency department [15]. Use of pre-existing Gene Xpert systems, primarily employed for tuberculosis diagnostics, could potentially also increase access to HIV-1 VL testing.

The need for increased volume of VL testing is expected to increase significantly in LMICs to meet the UNAIDS “90-90-90” monitoring to ensure undetectable VL in 90% of patients on antiretroviral therapy by 2020. Furthermore, in absence of an effective vaccine or cure for HIV/AIDS, optimizing the clinical management of HIV-1 infected individuals is important for population health [22]. Routine monitoring of HIV-1 VL is a key strategy for assessing risk of disease progression and guiding ART, both before and during antiretroviral therapy [8,23]. Quantification of HIV-1 RNA in plasma is an important marker to ensure successful treatment, identify early treatment failure, and determine whether a patient’s ART regime needs to be changed [8,15,16,21,23].

## Strengths and Limitations of this Study

To the best of our knowledge this is the first study to validate the Xpert-HIV-1 VL method in a Malian patient who represents the genetic backgrounds of much of West Africa’s populations. The study, nevertheless, has some limitations. The study did measure the genetic diversity of the HIV isolates tested, which may important because of the known inaccuracy the Xpert-HIV-1 VL assay in patients with high VLs of some HIV subtypes G and CRF02_AG strains [5,13,24]. The differences in performance between HIV-1 VL quantification assays are likely due to mutations in some subtypes, which impact the primer and probe binding sites targeted by these assays [5].

## Conclusion

Our findings showed that the Xpert-HIV-1 VL technique performs well as the two reference standard assays, the Roche TaqMan and Abbott m2000 RT. It has many features that are well suited to very limited resource settings, including its simplicity, rapid result time, and potential for existing GeneXpert technology to be deployed for for HIV-1 VL quantification. The results of this study show that Xpert HIV-1 VL can be an important tool in West Africa for optimizing HIV-1 infected patient management and help to accelerate and achieve the UNAIDS 90-90-90 global targets.

## Figures and Tables

**Table 1.** Clinical and demographic characteristics of the 138 patients included in this study.

Patients Characteristics (N=138)	Number (n)	Frequency (%)
**Sex**		
Male	43	31.2
Female	95	68.8
**Age (years)**		
0–17	10	7.2
18–35	51	37
36–50	46	33.3
>50	31	22.5
**CD4 count (cells/mL)**		
≤ 200	15	10.9
201–350	8	5.8
351– 500	7	5.1
> 500	32	23.2
Unknown	76	55
**Receiving antiretroviral medication**		
No treatment	28	20.29
NRTI + NNRTI	94	68.12
NRTI + PI	14	10.14
NRTI + PI + II	2	1.45

**Table 2a.** Agreement in HIV-1 Viral load quantification between Xpert HIV1-VL and Roche TaqMan according to the threshold of the two assays.

Variables		Roche TaqMan		
		Quantified	Not Detected	Total
Xpert® HIV1-VL	Quantified	54	2	56
	Not Detected	4	65	69
	Total	58	67	125

**Table 2b.** Performance, utility and reliability/reproducibility of Xpert HIV1-VL assay compared to the Roche TaqMan assay.

Statistics	Estimation	95% CI
Accuracy (%)	95.2	89.92–97.78
Sensibility (%)	93.1	83.57–97.29
Specificity (%)	97.01	89.75–99.18
Positive Predictive Value (%)	96.43	87.88–99.02
Negative Predictive Value (%)	94.2	86.02–97.72
Positive Likelihood Ratio	31.19	11.67–83.33
Negative Likelihood Ratio	0.07109	0.04351–0.1161
Diagnostic Odds Ratio (DOR)	438.8	77.37–2488
Cohen’s Kappa	0.9	0.72–1.07

**Table 3a.** Agreement in HIV-1 Viral load quantification between Xpert HIV1-VL and Abbott m2000RT according to the threshold of the two assays.

Variables		Abbott m2000 RT		
		Quantified	Not Detected	Total
Xpert® HIV1-VL	Quantified	57	6	63
	Not Detected	4	69	73
	Total	61	75	136

**Table 3b.** Performance, utility and reliability/reproducibility of Xpert HIV1-VL test compared to the Abbott m2000 RT reference test.

Parameters	Estimation	95% CI
Accuracy (%)	92.65	86.99–95.96
Sensibility (%)	93.44	84.32–97.42
Specificity (%)	92	83.63–96.28
Positive Predictive Value (%)	90.48	80.74–95.56
Negative Predictive Value (%)	94.52	86.74–97.85
Positive Likelihood Ratio	11.68	8.405–16.23
Negative Likelihood Ratio	0.07128	0.04356–0.1166
Diagnostic Odds Ratio (DOR)	163.9	44.09–609.1
Cohen’s Kappa	0.85	0.68–1.02
